# Comparing Automated Morphology Quantification Software on Dendrites of Uninjured and Injured *Drosophila* Neurons

**DOI:** 10.1007/s12021-021-09532-9

**Published:** 2021-08-03

**Authors:** Carolee Nguyen, Katherine L. Thompson-Peer

**Affiliations:** 1 Department of Developmental and Cell Biology, University of California, Irvine, Irvine, CA 92697 USA; 2 Reeve-Irvine Research Center, University of California, Irvine, Irvine, CA USA; 3 Center for the Neurobiology of Learning and Memory, University of California, Irvine, Irvine, CA USA

**Keywords:** Dendrites, Dendrite arbor, *Drosophila*, Dendrite regeneration, Automated analysis, Software comparison, Dendrite injury

## Abstract

Dendrites shape inputs and integration of depolarization that controls neuronal activity in the nervous system. Neuron pathologies can damage dendrite architecture and cause abnormalities in morphologies after injury. Dendrite regeneration can be quantified by various parameters, including total dendrite length and number of dendrite branches using manual or automated image analysis approaches. However, manual quantification is tedious and time consuming and automated approaches are often trained using wildtype neurons, making them poorly suited for analysis of genetically manipulated or injured dendrite arbors. In this study, we tested how well automated image analysis software performed on class IV *Drosophila* neurons, which have several hundred individual dendrite branches. We applied each software to automatically quantify features of uninjured neurons and neurons that regenerated new dendrites after injury. Regenerated arbors exhibit defects across multiple features of dendrite morphology, which makes them challenging for automated pipelines to analyze. We compared the performances of three automated pipelines against manual quantification using Simple Neurite Tracer in ImageJ: one that is commercially available (Imaris) and two developed by independent research groups (DeTerm and Tireless Tracing Genie). Out of the three software tested, we determined that Imaris is the most efficient at reconstructing dendrite architecture, but does not accurately measure total dendrite length even after intensive manual editing. Imaris outperforms both DeTerm and Tireless Tracing Genie for counting dendrite branches, and is better able to recreate previous conclusions from this same dataset. This thorough comparison of strengths and weaknesses of each software demonstrates their utility for analyzing regenerated neuron phenotypes in future studies.

**Supplementary Information:**

The online version contains supplementary material available at 10.1007/s12021-021-09532-9.

## Introduction

The complex architecture of neurons are composed of highly branched dendrites extending from the cell body and a long axon projecting to target cells. The primary function of dendrites is to receive information from the environment or from upstream neurons and to integrate input signals across the dendrite arbor. Despite their importance, only recently have researchers begun testing the regenerative capacity of dendrites after injury (Song et al., 2012; Stone et al., 2014; Thompson-Peer et al., 2016). Dendrites can be injured in various manners including stroke, traumatic brain injury, and neurodegenerative diseases (Gao et al., 2011; Klapstein et al., 2001). Subsequently, dendrite regeneration is affected by environmental and cellular factors that differ across neuron types and forms of injury. Such circumstances would be expected to create variability in the resulting morphologies of individual regenerated neurons. In order to understand the cellular mechanisms involved in dendrite regeneration, it is necessary to investigate changes in neuron morphologies after injury.

Dendrite regeneration can be assessed by tracing the neuron’s architecture. Neuronal tracing, a process which determines the shape and location of axons and dendrites in respect to the cell body of a neuron, is a computational technique frequently utilized to analyze neuron morphologies. Common parameters used to investigate neuronal phenotypes are total dendrite length and number of dendrite branches, which can reveal changes in dendrite architecture throughout development (Henley et al., 2019). Tracing neurons allows neuroscientists to digitally quantify regeneration and understand how different types of injuries affect overall dendrite architecture. However, tracing regenerated neurons is difficult because newly formed dendrite branches are disorganized, exhibit self-avoidance defects, and have a denser arbor compared to dendrites of wildtype uninjured neurons (Thompson-Peer et al., 2016). This issue is further complicated by the fact that many existing tracing software have been specifically developed and used to quantify healthy, uninjured neurons (Donohue & Ascoli, 2011).

A common technique for dendrite analysis involves hand tracing neurons using the Simple Neurite Tracer plug-in of ImageJ software (Longair et al., 2011; Rueden et al., 2017). This semi-manual approach involves identifying the beginning and end points of dendrites and digitally drawing individual branch segments throughout an entire neuron. Previous studies have manually quantified dendritic morphologies to investigate the cellular mechanisms involved in promoting dendrite development and regeneration. For example, Jiang et al. (2019) examined the role of epidermal somatosensory neurite ensheathment on neuron morphogenesis by hand tracing specific classes of nociceptive sensory neurons (Jiang et al., 2019). Using a similar technique, DeVault et al. (2018) demonstrated that the regenerative capacity of dendrites decreases with age but can be compensated by inhibition of matrix metalloproteinase 2 (Mmp2) in surrounding tissue (DeVault et al., 2018). A more recent study discovered a novel function of the receptor tyrosine kinase (RTK) orphan receptor (Ror) for promoting dendrite regeneration as well (Nye et al., 2020). While digital hand tracing remains a popular choice for analyzing neuronal phenotypes, this approach is laborious and is hampered by variability in how researchers distinguish individual dendrite branches (Donohue & Ascoli, 2011).

In order to aid with such tasks, many automated algorithms have been developed to address the challenges involved with neuronal tracing (Chen et al., 2015; Kanaoka et al., 2019; Myatt et al., 2012; Peng et al., 2010; Smafield et al., 2015). These software, which have largely been developed by independent studies, demonstrate the use of self-learning algorithms for particular issues at hand. Additionally, several studies have demonstrated the applicability of commercially available software as potential candidates for analyzing dendritic morphologies with minimal user input. Agostinone et al. (2018) reconstructed dendritic arbors to investigate whether an insulin supplement was capable of promoting new branch formation after axon-injury-induced retraction in retinal ganglion cells (RGCs) (Agostinone et al., 2018). Tapias et al. (2013) studied the effects of neurodegeneration on dendrite morphologies by quantifying neurons subjected to neurotoxic treatments (Tapias et al., 2013). The increase in availability of automated tracing software has undoubtedly helped to facilitate such analyses; however, there still remains a need for a standardized neuron tracing protocol.

A subset of peripheral sensory neurons in *Drosophila,* known as the multidendritic dendritic arbor (md-da) neurons, are often used to investigate dendrite development, in part because of their distinct morphology amongst specific classes (Grueber et al., 2002). *Drosophila* da neurons are categorized based on gene expression and morphology of their dendritic arbors, which vary in branching complexity across different classes (Jan & Jan, 2010). The dendritic arbor of class I da neurons are established in early larval development and have the most simple dendrite architecture. In contrast, class IV da neurons contain several hundred individual dendrite branches, which grow throughout development, making them the most complex class of md-da neurons. *Drosophila* da neurons are ideal for studying dendrite regeneration, because their morphology is highly stereotyped from animal to animal, neurons can be easily located across different imaging sessions, and their superficial location makes optical dendrite injuries straightforward (Song et al., 2012; Stone et al., 2014).

Several papers have examined various techniques of neuron reconstruction primarily on mammalian brain neurons (Acciai et al., 2016; Donohue & Ascoli, 2011; Halavi et al., 2012; Meijering, 2010; Parekh & Ascoli, 2013). However, these papers only compared specific features and methodologies of each software, and the accuracy of these programs have yet to be compared comprehensively to hand tracing or to one another when specifically addressing the unique challenges presented by dendrites regenerated after injury. Many automated image quantification software claim to be more efficient than manual hand tracing. Yet, their accuracies are still validated with the golden standard of hand tracing (Donohue & Ascoli, 2011). Given such context, developing a standardized procedure that automates neuron tracing with high accuracy will resolve a significant bottleneck in analyzing the complex arbor of regenerated dendrites.

In this study, we compared the accuracy and efficiency of various automated image analysis pipelines using the same data set in Imaris, which is commercially available (Bitplane), and DeTerm and Tireless Tracing Genie (TTG), which are both independently developed by researchers (Iyer et al., 2013; Kanaoka et al., 2019). We evaluated the accuracy of these software relative to the hand tracing technique, when applied to uninjured and regenerated *Drosophila* class IV da neurons. In order to streamline the process of neuron tracing, we quantified the duration it takes to accurately trace neurons using each software, which could potentially replace traditional hand tracing methods. We expect that one of these automated approaches will yield more accurate results than the others, closer to hand tracing, but will also be more efficient at analyzing neuron morphologies.

## Methods

### Image Acquisition

Class IV ddaC da neurons in *Drosophila* larvae were injured and imaged as previously described (Thompson-Peer et al., 2016). In this study, we re-analyzed the same data from that manuscript of the heterozygous cross progeny of w^1118^; ppk-CD4-tdGFP^1b^ (Han et al., 2011) adults crossed to w^1118^ adults. This fly line drives expression of the membrane-tagged CD4-tdGFP exclusively in the class IV da neurons of the *Drosophila* peripheral nervous system under the control of the cell-type specific *ppk* promoter. The ppk promoter is relatively strong, and quite specific, which results in an image with a good signal/noise ratio. Adult flies were allowed to lay eggs onto grape agar plates with a dot of wet yeast paste for a short period of time (approx 4 h), and the embryos were then allowed to develop and eventually hatch for the desired length of time (hours AEL) at room temperature. At the time of injury, animals were individually mounted onto agarose pads on slides, covered with glycerol and a coverslip, and imaged on a Zeiss LSM 580 microscope equipped with a Chameleon 2-photon laser at 930 nm. In a version of the injury assay that is a hybrid of the two-photon injury described in Song et al. (2012) and the total dendrite removal described in Stone et al. (2014), the two-photon laser was used to first image the membrane-tagged GFP in the neuron, then focused on the 2–5 branch points closest to the cell body, with higher power and slower scanning speed, to cut off all the dendrite branches of these neurons (so-called “balding” the neurons). In order to eliminate the complicating factor of adjacent neurons invading the territory, adjacent neurons were ablated when dendrites were injured at 24, 36, or 48 h AEL. Generally, neurons in segments T3, A2, A4, and A6 were ablated; neurons in segments A1 and A3 were balded; and the neuron in segment A5 remained as the uninjured age-matched control neuron. After injury, animals were housed individually on grape plates with yeast paste at room temperature, imaged at 24 h later and again 72 h later. Any injured neurons that showed a branch(es) that had been missed at 24 h after injury were not included in this analysis. Any neurons or animals that did not survive all the way through to the final imaging session were also not included in this analysis. At 24 h after injury (24 h AI for injured neurons and 24 h AMI for mock-injured neurons) and 72 h after injury and after mock injury A(M)I animals were individually mounted again on an agarose pad in glycerol under a coverslip, and imaged on a Leica SP5 confocal microscope using an HC PlanAPO 20x/0.75 IMM oil objective and standard 488 nm laser illumination. Later, after out of focus planes were removed, Z-stacks were converted to maximum intensity projections using ImageJ. Further processing of the images, such as background subtraction, was not performed.

Uninjured neurons @24 h after egg laying (AEL) at 24 h (*n* = 12) and 72 h after mock injury (AMI) (n = 12). Injured neurons @24 h AEL at 24 h (*n* = 8) and 72 h after injury (AI) (n = 8). Uninjured @36 h AEL at 24 h (*n* = 3) and 72 h AMI (n = 3). Injured @36 h AEL at 24 h (*n* = 6) and 72 h AI (n = 6). Uninjured @48 h AEL at 24 h (*n* = 15) and 72 h AMI (n = 15). Injured neurons @48 h AEL at 24 h (*n* = 16) and 72 h AI (n = 16). Uninjured @72 h AEL at 24 h (*n* = 10) and 72 h AMI (*n* = 11). Injured @24 h AEL at 24 h (*n* = 13) and 72 h AI (n = 13).

### ImageJ Measurements

Manual dendrite tracing was conducted using the Fiji distribution of Simple Neurite Tracer plug-in (Longair et al., 2011) in ImageJ software (using the most recent Fiji version of ImageJ (Schindelin et al., 2012) https://imagej.nih.gov/ij/). Using the previously defined protocol, individual dendrite branches of a neuron were traced from an acquired 2-D or converted 3-D image (Thompson-Peer et al., 2016). This plug-in allows users to quantify dendrite branch and length by tracing and registering individual branches with respect to the cell body. Individual dendrite fragments were selected to determine the beginning and end of each individual dendrite. This process was repeated for all dendrites in each neuron. Total dendrite branch number was extracted from ImageJ path to data output. Specifically, branch number is the number of terminal branch tips. Path lengths of individual dendrites were totaled to reveal the total dendrite length of the neuron.

### DeTerm Measurements

DeTerm source code and network model was run in Python (v3.6.8). Several external python packages, including tensor flow, scipy, scikit-image, numpy, and matplotlib were installed, as directed by the DeTerm supplementary protocol (Kanaoka et al., 2019). Input images for DeTerm were pre-processed in ImageJ: raw input images were acquired by inverting the lookup table (LUT) and region of interest (ROI) input images were acquired by manually selecting a ROI in ImageJ software for each original image in our dataset. DeTerm software was executed in the command line through a series of available python scripts (https://bitbucket.org/skibbe/determ/wiki/Home). Raw and ROI input images were processed in DeTerm to generate output images and positional data. Each generated output image was manually corrected by subtracting mis-detected dendrite branch terminals and adding undetected terminal points using the multipoint tool in ImageJ as false positives and negatives respectively. These points were removed or added from the original output of total branch terminals detected by DeTerm accordingly. Thus, DeTerm also quantifies the number of terminal branch tips.

### Imaris Measurements

Imaris software (ver. 9.3.1–9.5.0, Oxford Instruments) provided by the UC Irvine Optical Biology Core facility was used for image analysis. Neuron images were imported into Imaris software as flat 2-D maximum intensity projection images to avoid inappropriate z-direction terminal branches and to make comparable analyses to the other 2-D software tested. Image processing was performed by adjusting threshold levels to remove background noise for each image. Images were cropped within Imaris to exclude unwanted neighboring neurons. Neuron reconstruction was performed using automated detection by the Filament Tracer tool. The largest and thinnest diameters of the neuron were manually determined to generate dendrite starting and seed points. The thresholds for these points were adjusted in order to cover missed regions on the neuron of interest, in which the automated filament was generated. Small dendrite branches were reconstructed as though they were dendritic spines. The generated filament was edited in the creation wizard window to correct mis-detected and undetected branches. The semi-automated technique for neuron reconstruction was used to manually add undetected branches. Although Imaris is capable of counting either total number of dendrite segments (counting primary branches as separate from secondary branches, and so forth), to produce data that is comparable to the other algorithms, we only report here the number of terminal dendrite tips (marked as total branch number).

### Tireless Tracing Genie Measurements

Tireless Tracing Genie plug-in was installed and ran on ImageJ software. An inverted ROI was selected in order to exclude unwanted neighboring neurons. Individual values of the neuron skeleton after processing were added using the Cox Sums program provided (Iyer et al., 2013). Instead of directly measuring dendrite length, this plug-in utilizes the number of slab voxels for each neuron skeleton as an equivalent parameter for total dendrite length. The pixel conversion factor (pixels to microns) was obtained from ImageJ for each individual image to manually convert the number of slab voxels to total dendrite length in microns. TTG uses the number of end point voxels as an equivalent parameter for total dendrite branch number, thus also counting the number of terminal branch tips.

### Time Calculations

The time required to trace individual neurons was recorded for a handful of neurons analyzed through each pipeline. Pre-processing times included the time required to select ROIs, adjust brightness and contrast, and apply other image processing features. Post-processing times included the time required to manually edit and correct each image for inaccuracies after processing individual images through each pipeline. The times required in each pre-processing and post-processing step were recorded and added together to sum a total average time for each automated software. Each pipeline varied in the amount of pre-processing and post-processing required which was noted and added when averaging the computing duration for neuron reconstruction. The average tracing times of each software tested was compared to hand tracing. This process was repeated for a small subset of neuron images (*n* = 10 neurons; *n* = 5 neurons mock injured at 24 h AEL then imaged at 24 or 72 h AMI; n = 5 neurons bald or mock injured at 48 h AEL then imaged at 24 or 72 h A(M)I). Tireless Tracing Genie was not included in these time calculations as the time it takes the pipeline to analyze each image was nearly instant and did not output images for manual correction.

### Statistical Analysis

The same 124 images were imported and analyzed in each software to obtain parameters of total dendrite branches and total dendrite length. These 124 images represent neurons across conditions of 24 h, 36 h, 48 h, and 72 h after egg laying (AEL), and imaged at 24 h and 72 h after injury (AI) or after mock injury (AMI) as described. Averages ± standard deviation error bars are shown throughout the manuscript. The statistical significance of total dendrite branch number amongst three pairs of methods (ImageJ vs DeTerm, ImageJ vs Imaris, and DeTerm vs Imaris) was determined using paired two-sample t-tests (*p* ≤ 0.05). In order to compare the results between ImageJ, DeTerm, and Imaris, the statistical significance of total dendrite branches was determined using a one-way ANOVA test followed by Tukey’s multiple corrections test, in Prism 8 (GraphPad, San Diego, CA). Biostatistical tests were determined in consultation with the UCI Institute for Clinical & Translational Science resources for Biostatistics, Epidemiology, and Research Design. Total dendrite length between two pairs of methods (ImageJ vs Imaris and ImageJ vs Tireless Tracing Genie) was determined using paired two-sample t-tests (p ≤ 0.05). The average tracing times of ImageJ, DeTerm, and Imaris was compared using a one-way ANOVA test followed by Tukey’s multiple corrections test, as previously mentioned.

## Results

### DeTerm Requires Significant Manual Correction, but Eventually Counts Dendrite Tip Number Accurately

**Fig. 1:**
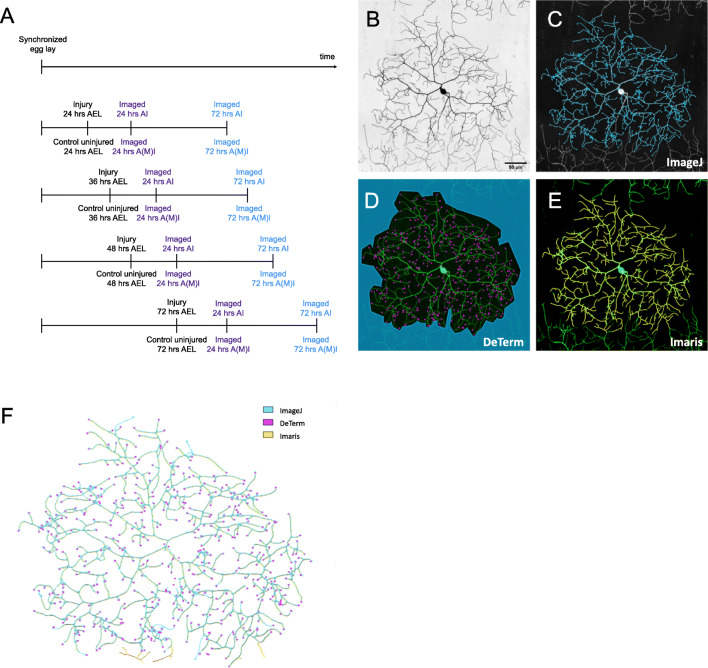
Timeline of the complete data set that is reiteratively processed through data analysis pipelines. **A** Timeline for experiments. After a synchronized egg lay, neurons are injured (or not injured, in the case of control uninjured neurons) at 24, 36, 48, or 72 h after egg laying (AEL). Animals are then recovered, and continue to develop. Neurons are imaged at 24 h after injury (AI) or after mock injury (for uninjured neurons, AMI) and again at 72 h AMI. **B** Representative image of an injured & regenerated neuron, balded at 24 h AEL and imaged at 72 h AI. **C** ImageJ hand tracing analysis of the neuron in panel **B**. **D** DeTerm analysis of neuron in panel **B**. Purple dots are branch tips counted by DeTerm before manual correction. Blue shading indicates the area marked as outside the dendrite arbor. **E** Imaris reconstruction of neuron in panel **B**. **F** ImageJ (blue) hand tracing analysis overlayed above DeTerm terminal branch detection (pink) output and Imaris neuron reconstruction (yellow). Branches traced by both ImageJ and Imaris appear green

Our dataset consisted of 124 images of ddaC peripheral nervous system neurons within abdominal segments A1-A6 in *Drosophila* larvae that came from 16 different conditions (Thompson-Peer et al., 2016). The dendrites of these neurons were either uninjured or injured using a two-photon laser injury method as previously described (Thompson-Peer et al., 2016). The conditions in our dataset are as follows: for injured neurons, dendrites were removed using a two-photon laser at 24 h, 36 h, 48 h, or 72 h after egg laying (AEL). Control neurons are uninjured neurons from these same animals. At 24 h AEL, 36 h AEL, and 48 h AEL, adjacent neurons were ablated, to reduce invasion of territory from adjacent uninjured neurons. Neurons are then imaged at both of two different timepoints: 24 h after injury (AI) or after mock injury (AMI) and 72 h (AMI). Thus, 4 ages × 2 treatment options (uninjured or injured) × 2 imaging time points (24 h A(M)I and 72 h A(M)I) results in the 16 conditions represented here (Fig. 1A).

As *Drosophila* larvae age, they grow in size, and the territory that each individual neuron is responsible for covering with its dendrite arbor proportionally increases in size as well. The youngest neurons have much smaller dendrite arbors, with much thinner dendrite branches, than the older neurons. For our data set, all images were collected with the same microscope and the same objective. However, the digital zoom is greater for the smaller, younger neurons than for the larger, older neurons, since the size of those neurons is smaller. Each neuron was imaged with a digital zoom that allowed the dendrite arbor to be captured in a Z-stack of a single 1024 pixel × 1024 pixel field of view (without stitching of adjacent images). Thus, while the younger neurons are smaller, and their dendrite branches are thinner, their dendrites are not captured by fewer pixels on the PMT detector of the confocal microscope. After removing Z-planes above or below the neuron of interest, the maximum intensity projection was generated (Fig. 1B).

All 124 neurons had been laboriously hand-traced using the Simple Neurite Tracer (SNT) plug-in in ImageJ (Fig. 1C). Hand tracing quantification is labeled as “ImageJ” throughout the study. We had measured the number of terminal dendrite tips (annotated as total branch number) and the total dendrite length of all branches summed together.

**Fig. 2:**
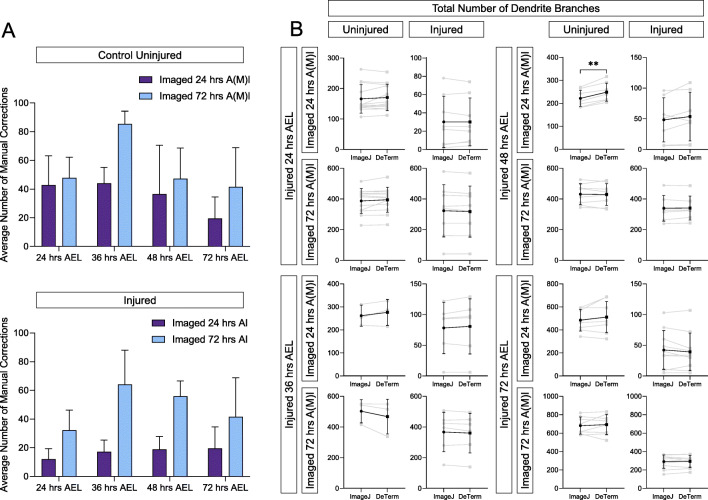
With significant manual correction, DeTerm generally counts the correct number of dendrite branch tips, but does not measure total dendrite length. **A** Average number of manual corrections required for neurons at each stage, whether uninjured (top) or injured (bottom), imaged 24 h A(M)I (blue) and 72 h A(M)I (gray), ± standard deviation error bars. **B** For neurons either injured at 24 h AEL, 36 h AEL, 48 h AEL, or 72 h AEL or uninjured controls, imaged at 24 h A(M)I (after injury or after mock injury) or 72 h A(M)I, the total number of branches counted by hand tracing in ImageJ or DeTerm is shown. Individual neurons are shown in gray (line connects the quantification of the same neuron), average ± standard deviation error bars are in black. ** *p* < 0.01 by paired t-test

We ran the complete dataset of 124 neurons through the DeTerm pipeline, a freely available software package which detects dendrite terminals based on a machine learning via artificial neural network algorithm (Kanaoka et al., 2019). DeTerm was trained by developers using a dataset of 70 wildtype class IV da (ddaC) neurons from wandering 3rd instar *Drosophila* larvae, where the dendrite tips had been manually annotated. After processing our data into the software, DeTerm generated output images of detected branch terminals which were then manually corrected to adjust for false positive and false negative points (Fig. 1D). Manual corrections were required at all time points and across all ages to accurately quantify the image data, including neurons uninjured at 72 h AEL that were imaged at 72 h AMI, which is most similar to the DeTerm training dataset (Fig. 2A). After manual correction, DeTerm resulted in similar counts of total dendrite branches compared to hand tracing across all conditions (*p* > 0.05, *n* = 124), excluding one time point (Fig. 2B). Automatic detection by DeTerm resulted in a statistically higher total count of dendrite branches in neurons uninjured at 48 h AEL that were imaged at 24 h AMI compared to hand tracing (*p* < 0.0001, 221 ± 35 dendrites from ImageJ versus 248 ± 39 dendrites for DeTerm, *n* = 8). Overall, with manual correction, DeTerm performs well for counting total branch number. However, as DeTerm does not measure dendrite length, we could not extract that parameter from our dataset using this pipeline.

### Imaris Reconstructs Arbors to Correctly Count Branch Number but Underestimates Dendrite Length

**Fig. 3:**
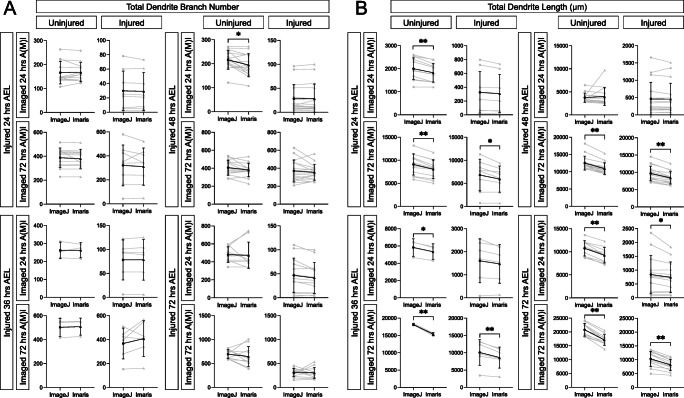
With manual correction, Imaris counts the correct number of dendrite branch tips, but significantly underestimates total dendrite length. **A** Total number of branches counted by hand tracing in ImageJ or by reconstruction in Imaris is shown. Individual neurons are shown in gray (lines connect the quantification of the same neuron), average ± standard deviation error bars are in black. None of the pairwise comparisons are significantly different. **B** Total dendrite length measured by hand tracing in ImageJ or by reconstruction in Imaris is shown. * *p* < 0.05, ** *p* < 0.01 statistically significant difference by pairwise t-test. Absence of an asterisk indicates no significant difference was observed

Next, we ran the complete dataset of 124 images through the commercially available software package Imaris (Oxford Instruments). Unlike DeTerm which only marks dendrite tips, Imaris reconstructs the entire dendrite arbor, allowing for quantification of a variety of features (Fig. 1E). Imaris counted similar numbers of total dendrite branches across all 16 conditions compared to hand tracing (p > 0.05, n = 124) (Fig. 3A). However, automatic detection by Imaris measured significantly shorter total dendrite lengths than hand tracing for 11 out of 16 ages and conditions (Fig. 3B). For example, in neurons uninjured at 24 h AEL that were reimaged at 24 h AMI, ImageJ measured an average length of 2002 ± 468.6 μm, compared to 1826 ± 406.5 μm for Imaris (p < 0.0001, n = 12). This significant under-measurement of dendrite length persisted in these same neurons when they were later re-imaged at 72 h AMI. At these time points, ImageJ and Imaris counted statistically similar numbers of dendrite branches. We wanted to ensure that consistent under-measurement of dendrite length was not simply a calculation error, so we measured the direct distance between two points on three images in Imaris and ImageJ for validation. Both software gave the same measurement length, suggesting that the observed difference in total arbor length is not merely a conversion error from pixels to microns (data not shown). Thus, after manual correction, Imaris is able to accurately count branch numbers, but significantly underestimates dendrite length in reconstructed arbors.

### Imaris is Slightly Better than DeTerm at Counting Branch Number

**Fig. 4:**
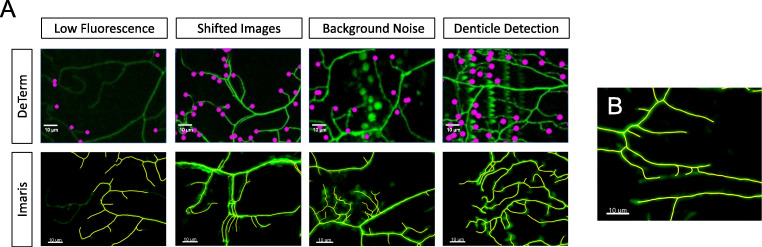
Manual correction of common errors in both DeTerm and Imaris. **A** These errors include adding in areas of low fluorescence, accounting for animal movement during image collection, removing high background detection, and removing detection of the denticle belt. **B** Looking closely at the Imaris reconstructed arbor, inappropriate shortening of small dendrite branches may account for the under-measurement of total dendrite arbor length

One significant difference between a hand tracing approach and the automated approaches is the need for after-the-fact manual correction of automated image analysis performed by DeTerm and Imaris. Like DeTerm, Imaris also required extensive correction, though since this step is embedded within the pipeline, we were unable to count the number of corrections made. The types of neuron features that required manual correction were observed to be similar for both automated pipelines (Fig. 4A). In cases where the fluorescent signal for neurons was poorly contrasted by a bright autofluorescent background, both DeTerm and Imaris excluded branches entirely, or only partially reconstructed the arbor (Fig. 4A). Autofluorescence from background structures such as the denticle belt could be misinterpreted as dendrites by automated approaches. If the animal moved slightly during image acquisition, which resulted in duplicating branches by a double shadow, the software would erroneously double count branches in the final projection image. Finally, automated approaches would trace the axon in many cases as well, since the proximal region of the axon is in a nearby Z-plane. In these cases, the axon was manually removed and not included as part of dendrite architecture. Manual correction allows users to remedy these errors where branches are either over- or under-counted by automated pipelines, that of which would be correctly traced by hand. Having manually corrected the Imaris reconstructions for dendrite branch number, we investigated potential reasons for the significant under-measurements of dendrite length in those reconstructed neurons. Upon closer inspection, only a number of small terminal branches were partially captured (Fig. 4B). Presumably, if enough branches are incompletely captured, this may subtract from the total length of the dendrite arbor observed while maintaining accurate values of total dendrite branches.

**Fig. 5:**
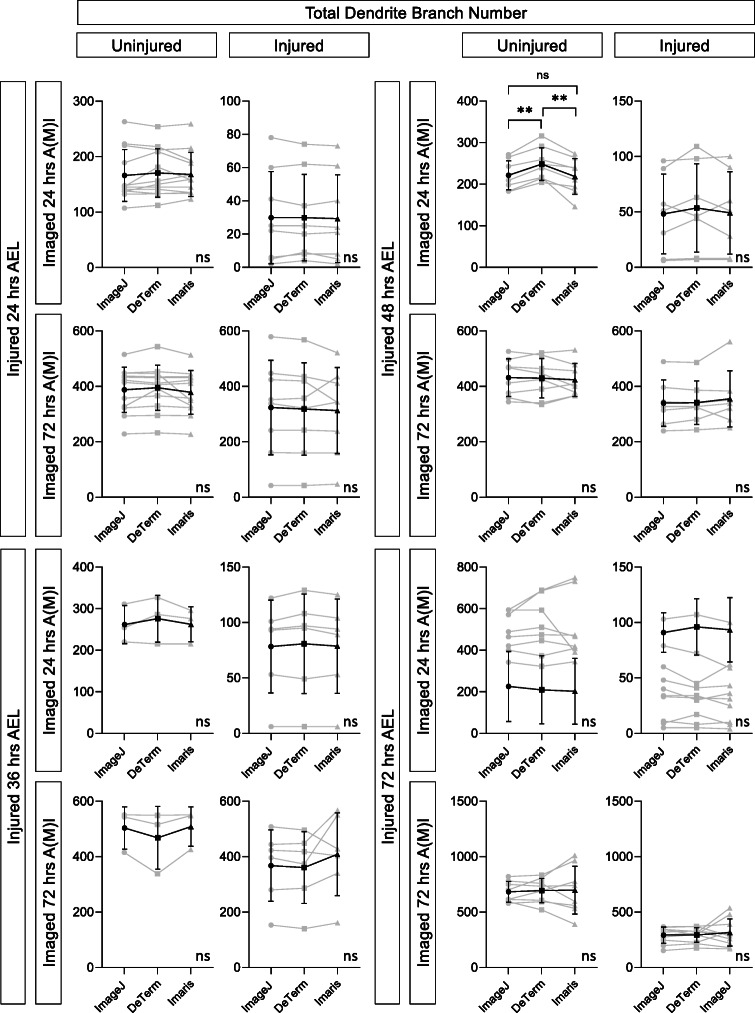
DeTerm and Imaris both generally count the correct number of dendrite branches on average, though DeTerm overcounts in one condition. Total number of branches counted by hand tracing in ImageJ or by automated analysis in DeTerm or by reconstruction in Imaris is shown. Individual neurons are shown in gray (lines connect the quantification of the same neuron), average ± standard deviation error bars are in black. ** *p* < 0.01 by one-way ANOVA with Tukey’s multiple corrections test, otherwise no statistical difference was found

Since DeTerm does not measure total dendrite length, we could not compare this parameter across all tested approaches. However, each automated approach was able to extract values of dendrite branch number. DeTerm and Imaris resulted in similar counts of total dendrite branches compared to hand tracing across all injury conditions (*p* > 0.05, *n* = 124), except for one (Fig. 5). DeTerm counted significantly more dendrite branches than ImageJ and Imaris in control uninjured neurons at 48 h AEL and imaged at 24 h AMI (*p* = 0.0019 compared to ImageJ, *p* = 0.0007 compared to Imaris, *n* = 8). For this same condition, DeTerm averaged a greater number of total dendrite branches (μ = 248.5 ± 39.2 dendrites) compared to ImageJ (μ = 221.6 ± 35.0 dendrites) and Imaris (μ = 218.6 ± 43.1 dendrites). For the remaining 15 conditions, there were no significant differences observed amongst ImageJ, Imaris, and DeTerm for counting dendrite branch number (*p* > 0.05).

Do the different algorithms perform differently on simpler versus more complex arbors? We compared each algorithm against hand-tracing by calculating the relative error output for each neuron compared to hand tracing, and then normalized that to the number of branches for that neuron (Fig. S3). For example, if ImageJ counted 200 branches on a given neuron, but Imaris counted 210 branches, the relative error would be (200–210)/200, or -5% for that neuron. For uninjured control neurons, DeTerm’s relative error is sometimes positive (when it underestimates the number of branches) and sometimes negative (when it overestimates the number of branches), while Imaris’ relative error is usually positive, but both are generally small. However, the size of the relative error for both DeTerm and Imaris is larger and more variable when calculated for neurons regenerating after injury (Fig. S3B). This supports our assertion that the automated pipelines perform more like hand tracing on uninjured control neurons, but that quantification of regenerated neurons after injury is a greater challenge for these software.

Due to the dependence of researchers to identify every starting and end point of individual dendrites, it took an average of 21 min to hand trace individual neurons from our dataset (Table 1). DeTerm and Imaris, which required both preprocessing and postprocessing steps, added to the amount of time on each image amongst automated approaches. DeTerm averaged about 7 min to process each image including preprocessing and postprocessing, which is significantly quicker than manual hand tracing by ImageJ (*p* < 0.0001, *n* = 10). Similarly, Imaris required around 5 min per each image, which was significantly quicker than ImageJ as well (p < 0.0001, n = 10). However, both DeTerm and Imaris were not significantly quicker than each other (p > 0.05, n = 10). As previously mentioned, Tireless Tracing Genie was not included in these time calculations as the time it takes the pipeline to analyze each image was nearly instant. Since this approach did not output images for manual correction, we could not compare post processing times against the other software tested.

**Table 1:** Average Tracing Times (*n* = 10). Hand tracing takes significantly longer than Imaris or DeTerm

Software	Pre-processing Time (min:sec. 1/100 s)	Tracing Time (min:sec. 1/100 s)	Post-processing Time (min:sec. 1/100 s)	Total (min:sec. 1/100 s)
ImageJ	N/A	20:58.4	N/A	20:58.4
DeTerm	01:05.7	01:52.3	04:06.3	07:09.5
Imaris	00:30.0	01:48.8	02:41.0	04:29.8

Pre-processing, automated tracing time, and manual editing time are shown for the same 10 neurons to go through ImageJ, DeTerm, and Imaris. These neurons were not chosen to be representative of all 16 conditions, but the relative time involved should be comparable across approaches

### Tireless Tracing Genie Underestimates Dendrite Branch Number and Overestimates Dendrite Length

**Fig. 6:**
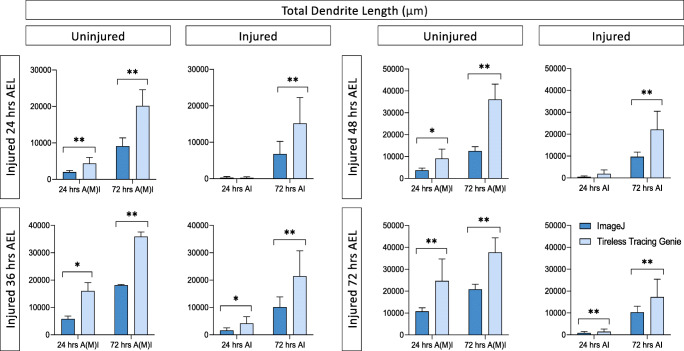
Tireless tracing genie consistently overestimates dendrite length. Average total dendrite length measured by hand tracing in ImageJ or by Tireless tracing genie is shown ± standard deviation. * *p* < 0.05, ** *p* < 0.01 statistically significant difference by pairwise t-test. Absence of an asterisk indicates no significant difference was observed.

Unlike DeTerm and Imaris, Tireless Tracing Genie does not offer the function to view processed images for manual correction. Due to this, we were unable to manually edit the analysis of dendrite architecture to add missed branches, remove inappropriate branches, or extend partially traced branches. We examined two output parameters in order to determine the total number of branches: *branches* and *end point voxels*, which is equivalent to the number of endpoints. The *branches* output reports the number of branch segments, so a single dendrite branch may have many segments, which is not comparable to the ImageJ branch tip number. The *end point voxels* output significantly underestimated the number of branches, and varied across all conditions (Fig. S1). Total dendrite length was extracted from Tireless Tracing Genie as *total slab voxels*, which the developers reported as nearly equivalent to total length. We individually converted each output length from pixels to microns, in order to determine total dendrite length in microns. After doing so, we found that total dendrite length was overestimated in nearly all cases except two (Fig. 6).

### Only Imaris, Among All the Automated Approaches, Reproduced Essential Conclusions of ImageJ Manual Analysis

This data set is a subset of the experiments generated for Thompson-Peer et al., 2016, where conclusions from the manual analysis were first described. At any age, branch number and branch length is less at 24 h after injury compared to uninjured neurons. There were four primary conclusions from this subsection of the data in that manuscript when neurons were imaged 72 h after injury, depending on the age at the time of injury (Table 2). For neurons injured at 48 h AEL, when they were imaged at 72 h AI, dendrite branch number had regenerated enough to not be significantly different from uninjured age-matched controls, but total dendrite length remained significantly shorter. For neurons injured slightly later in development, at 72 h AEL, when they were imaged at 72 h AI, dendrite branch number and total dendrite length were both significantly less than age-matched uninjured controls. We performed these same comparisons on the data as quantified by DeTerm (Fig. S2A), Imaris (Fig. S2B), and Tireless Tracing Genie (Fig. S2C), summarized in Table 2.

**Table 2:** Comparison to Thompson-Peer et al. (2016). Automated pipelines compared to manual tracing in their ability to detect major similarities and differences between uninjured and regenerated neurons

Hours AEL	Observation	ImageJ	DeTerm	Imaris	Tireless Tracing Genie
Injured 48 h AEL	At 24 h A(M)I, injured neurons have fewer branches than uninjured neurons	**	**	**	*
	At 72 h A(M)I, injured neurons have fewer branches than uninjured neurons	ns	*	ns	ns
	At 24 h A(M)I, injured neurons have shorter total length than uninjured neurons	**	n/a	**	**
	At 72 h A(M)I, injured neurons have shorter total length than uninjured neurons	**	n/a	**	**
Injured 72 h AEL	At 24 h A(M)I, injured neurons have fewer branches than uninjured neurons	**	**	**	ns
	At 72 h A(M)I, injured neurons have fewer branches than uninjured neurons	**	**	**	ns
	At 24 h A(M)I, injured neurons have shorter total length than uninjured neurons	**	n/a	**	**
	At 72 h A(M)I, injured neurons have shorter total length than uninjured neurons	**	n/a	**	**

Each approach was tested in their ability to recreate the major conclusions of how injury alters dendrite architecture. Neurons were (mock) injured at 48 or 72 h AEL, then at 24 h and 72 h later, mock uninjured control neurons were compared to injured neurons. Among the 3 automated pipelines, Imaris best supports biological conclusions similar to what is seen for ImageJ. * *p* < 0.05 statistical difference detected, in the direction indicated by the statement. ** *p* < 0.01 statistical difference detected, in the direction indicated by the statement. NS: no statistical difference was found. N/A: quantification is not an available output of the software. See also Fig. S2

At 24 h after (mock) injury, the differences between recently injured versus uninjured neurons are striking and should be obvious by any method of quantification. Only Imaris detected the obvious decrease in dendrite number and total length in neurons imaged 24 h after injury compared to age-matched uninjured controls. DeTerm detected the decrease in dendrite number but does not measure branch length, and Tireless Tracing Genie detected the decrease in length but failed to detect the decrease in branch number (in neurons injured at 72 h AEL, and detected the decrease in neurons injured at 48 h AEL with less significance than ImageJ hand tracing).

By 72 h after injury, injured neurons have regenerated significantly, but still fall short of uninjured control neurons in many important ways. DeTerm produced a novel slight but significant decrease in branch number of neurons injured at 48 h AEL, and recapitulated the same significant decrease in branch number of neurons injured at 72 h AEL; as DeTerm does not measure dendrite arbor length, it was unable to support conclusions about differences in dendrite length that were in the original manuscript. Imaris was able to successfully replicate all the findings previously observed with ImageJ. Tireless Tracing Genie detected the decreases in branch length for neurons injured at both 48 h AEL and 72 h AEL, relative to uninjured age-matched controls. While TTG replicated the finding that branch number regenerates to match age-matched controls in neurons injured at 48 h AEL, it failed to detect the significant impairment in branch number regeneration in neurons injured at 72 h AEL.

Overall, DeTerm would have allowed us to come to the same conclusions about branch number, but would not have been able to provide any insight into branch length. Tireless Tracing Genie would not have allowed us to come to the same conclusions about branch number, and significantly overestimates dendrite length. While Imaris underestimates dendrite length, this is consistent enough that it would have supported the same conclusions we came to in our earlier manuscript, while offering significantly faster data quantification.

## Discussion

While many automated software exist to aid with neuron tracing, different neuron types present various challenges for these software to adapt to. Our results highlight how each software analyzes morphological differences in dendrite architecture between wildtype uninjured and regenerated neurons. Since the morphology of an elaborate dendrite arbor determines how it functions as a receptive structure, it is important to examine how automated approaches capture the subtle differences caused by injury. Analyzing changes in dendrite morphology can help researchers identify cellular mechanisms involved in regeneration of dendrite architecture. As the field of dendrite regeneration continues to grow, the development of a reliable automated tracing software will be highly valued.

In this study, we compared how three publicly available automated neuron tracing software performed, both on uninjured and regenerated class IV *Drosophila* neurons. We evaluated the performance of DeTerm, Imaris, and Tireless Tracing Genie to accurately and efficiently quantify total dendrite length and number of dendrite branches. We determined that both DeTerm and Imaris counted a similar number of dendrite branches, though a great extent of manual correction was required. Tireless Tracing Genie significantly underestimated the total number of dendrite branches across all conditions. Unfortunately, none of the software we tested were capable of accurately extracting total dendrite length even following manual correction. DeTerm currently does not extract total dendrite length from images, and therefore, could not be compared to the other software. Tireless Tracing Genie overestimated length in almost every condition, despite proper conversion of pixels to microns. In contrast, Imaris significantly underestimated dendrite length in a vast majority of cases. Underestimations similar to that seen in Imaris have been previously reported by other studies. Meijering et al. (2004) observed underestimation of total dendrite length in a semi-automated approach, likely due to the algorithm shortcutting sharply bending segments (Meijering et al., 2004). Similarly, Smafield et al. (2015) attributed their underestimation of total dendrite length to disregard of dim branches and reciprocal overestimation of dendrite length in other parts of the neuron (Smafield et al., 2015). As shown in our study, automated reconstruction of dendrite branches by Imaris only captured a number of small terminal branches, which may be attributed to similar reasons as those seen in previous studies.

While none of the automated approaches are a perfect quantification of the dendrite arbor, limitations in reliability due to misquantification may be offset by increases in efficiency. The average neuron tracing time was determined by adding the time it took to prepare each image before tracing and the time it took for the software to trace both manual and automated approaches. Hand tracing by ImageJ took the longest time with an average of 21 min per image. DeTerm was significantly quicker than ImageJ with an average tracing time of 7 min. Likewise, Imaris’ average tracing time of 5 min was quicker than ImageJ, but was not significantly quicker than DeTerm. Our sample size consisted of mostly uninjured neurons imaged at 24 h AMI, which had the most simple arbors across all our conditions, and are certainly simpler than their injured counterparts and neurons in older animals. Regenerated neurons have disorganized branches, exhibit self-avoidance defects, and have denser arbors after injury compared to uninjured neurons. These defects exhibited by regenerating dendrites make analysis more complicated compared to uninjured neurons. Thus, the analysis times that we reported represent the shortest possible analysis time with each approach. Analysis of more complex arbors in older animals and an increased number of dendrite branches would expect to take proportionally longer with each approach.

DeTerm’s automated detection of dendrite branch number performed better in its original study compared to our study relative to manual tracing (Kanaoka et al., 2019). The difference in the results obtained from Kanaoka et al. (2019) and this study may be attributed to the original dataset used to train DeTerm’s artificial neural network. Their dataset consisted of 70 wildtype class IV da neurons of wandering 3rd instar *Drosophila* larvae while ours contained images of uninjured and injured neurons acquired at earlier time points. Thus, it is difficult to determine DeTerm’s applicability to younger animals or on regenerated neurons as we tested in this study. Wandering 3rd instar larvae are at least 96 h AEL compared to the larvae examined in this study, which are between 24 and 72 h AEL. Class IV da neurons grow throughout larval development, therefore the number of dendrite terminals detected by DeTerm in wandering 3rd instar larvae in the original study is much higher than the number we report here for younger neurons. Additionally, DeTerm was originally applied to neurons of varying nutritional conditions, which altered dendrite morphology, whereas the two-photon laser injury assay was used in our study. Differences in the intensity of each injury method may have resulted in varying regenerated morphologies. This may explain why DeTerm performed better at quantifying dendritic branches in wildtype as well as neurons subjected to minor injuries but struggled to accurately quantify complex regenerated dendrite arbors without manual correction.

Similarly, the original Tireless Tracing Genie study detected several thousand branches on average for each genetic condition analyzed and did not apply the program to simpler uninjured neurons utilized in our dataset (Iyer et al., 2013). Our results show that Tireless Tracing Genie significantly overestimates total dendrite length in almost every condition, making it difficult to assess its applicability for this purpose. Tireless Tracing Genie was originally used to quantify dendrite morphology of neurons in various genetic mutants, which had a greater number of dendrite branches and total arbor length compared to our samples. Given such, our results are not simply due to errors in the application, but rather due to differences in the morphologies of neurons utilized in each dataset.

Out of the three automated approaches tested, Imaris best suits our goal to study dendrite regeneration on both uninjured and regenerated dendrites. Our results demonstrate that purely automated methods do not yield accurate results, and manual correction is required to correct errors in resulting output traces. Imaris mitigates this issue by incorporating a strategy that combines automated reconstruction and user editing, through a semi-manual construction method similar to Simple Neurite Tracer. In contrast, DeTerm requires users to manually correct for missed and overcounted dendrite branches using an external software. While this step greatly enhances the accuracy of DeTerm, it also increases the amount of time required to effectively trace each image, which is important to consider when quantifying large datasets. Tireless Tracing Genie, while simple to install and execute, could not output annotated images from input images, making it difficult to assess the validity of the software’s performance. The choice of tracing method is an essential element in optimizing efficiency. DeTerm is operated via the command line, and thus requires knowledge on setting up programming environments, installing external libraries, and running Python scripts. On the other hand, Imaris’s user interface has a creation wizard that guides users through the tracing pipeline step-by-step. In addition, users can choose between various tracing strategies ranging from manual to automatic, and given such, Imaris’ customizable user interface could be considered more user-friendly. Moving forward, user-interface and program features will be significantly important to maximize the efficiency of automated neuron tracing software for studying dendrite regeneration.

While this study only tested three applicable software for quantifying regeneration in *Drosophila* neurons, we acknowledge that other image analysis techniques exist for this particular issue at hand. Sheng et al. (2019) and Satoh et al. (2012) both utilized a combination of time lapsed video imaging with image analysis software to observe and quantify the development of uninjured and regenerated *Drosophila* neurons, respectively (Satoh et al., 2012; Sheng et al., 2019). Many studies have utilized the commercial software, Neurolucida (MBF Bioscience), for neuron image analyses as well (Dickstein et al., 2016; Egger et al., 2012; Ghosh et al., 2011; Sohn et al., 2016). Previous studies have also identified the need for automated analysis of complex neuron morphologies. Similar to DeTerm, Soltanian-Zadeh et al. (2019) developed an algorithm based on a convolutional neural network (CNN) architecture for neuron image segmentation (Soltanian-Zadeh et al., 2019). While these studies demonstrate the applicability of machine learning software for neuron image quantification, they have only been illustrated to be useful for their own unique dataset. This issue of applicability of algorithms to external datasets is widely investigated, and a software that could successfully be applied to various types of neurons is desired for the future. It is also important to note that these results may not fully apply to other neuronal systems, or the same neuronal systems visualized using different methods. Images with poorer signal-to-noise ratios will be harder to quantify automatically, such as imaging on other microscopes, or with dimmer fluorophores, or deeper dendrites that are farther from the imaging coverslip.

Unlike automated approaches, hand tracing requires researchers to determine the starting and end points of individual dendrites themselves. While this completely eliminates post-processing times, it significantly adds to the time spent directly analyzing each image. On the other hand, state-of-the-art automated neuron tracing approaches still require intensive manual correction after algorithmic processing (Peng et al., 2011). While automated approaches significantly reduce the amount of time to trace dendrites, the time dedicated to manual correction could potentially render this advantage impractical. In fact, the online neuron morphology database Neuromorph.org primarily consists of neuron reconstructions using manual approaches most likely due to this reason (Ascoli et al., 2007). Therefore, it is important to consider that faster analyses may not necessarily be the most efficient. Additionally, pre-processing images by adjusting image quality and removing interfering signals can improve the performance of each software tested. Imaris allows users to adjust imaging settings within the software, while DeTerm and Tireless Tracing Genie require images to be pre-processed using external software, ImageJ. For Imaris, image processing was performed by adjusting threshold levels to remove background noise for each image. Images were then cropped within Imaris to exclude unwanted neighboring neurons. While the imaging settings were not altered for DeTerm and Tireless Tracing Genie, it is possible that their performances may improve with image quality. Increasing the neuron signal may have allowed DeTerm and Tireless Tracing Genie to detect dendrite branches that would have otherwise been undetected. Similarly, removal of background noise may reduce the instances in which branches are falsely misdetected by both software. It is important to consider all facets of automated techniques, as no software is going to perfectly quantify these features of a dendritic tree. The information we present here should help researchers in this cost/benefit analysis, to determine if the increase in efficiency afforded by automated pipelines compensates for the particular decrease in reliability of any individual measurement.

For this manuscript, we focused on extracting two specific measurements from these automated pipelines: branch number and total dendrite length. We focused on these parameters because they were important for the conclusions of our previous work, and because most of the software could deliver these quantifications, allowing us to cross compare the output results. However, these are only a few of the many important parameters that determine dendrite architecture. In addition to branch number and total length, other important parameters include dendrite branch order (the number of primary versus secondary versus terminal branches), branching location, and overlap with other branches of the same neuron (self-avoidance) or adjacent neurons (tiling). These features are frequently quantified using Sholl analysis and overlap measurements (O'Neill et al., 2015; Sholl, 1953). While Sholl analysis was not conducted or compared amongst the tested software in this study, the neuronal reconstructions of both ImageJ hand tracing and Imaris are capable of generating this valuable metric (along with a variety of other morphological parameters). Tireless Tracing Genie and DeTerm are not capable of supporting automated Sholl analysis. Thus, for questions where the Sholl analysis would prove useful, our conclusion that the Imaris pipeline best facilitates automated extraction of the features that can be manually extracted by hand tracing holds true. None of the approaches automatically measure crossing over (of other branches of the same neuron, as defects in self-avoidance, or of other branches of other neurons, as defects in tiling), but the measurements of dendrite length is necessary for the normalization of crossing over events per 1000 μm of dendrite length, and this data is reliably generated by ImageJ and Imaris (but not TTG nor DeTerm). This study presents a simplified analysis of the performance of several methods available for neuron tracing, which included parameters of total dendrite branches and total dendrite length, for studying dendrite architecture when comparing a wild-type to abnormal arbor.

## Information Sharing Statement

Most of the hand tracing analyzed in this manuscript was previously uploaded to NeuroMorpho.org, as part of the Thompson-Peer et al., 2016 manuscript. Any hand tracing not previously uploaded to the repository as part of the prior publication will be added to that repository.

DeTerm and TTG were previously published by their developers (Iyer et al., 2013; Kanaoka et al., 2019). Imaris is available commercially from Oxford Instruments.

## Supplementary Information

ESM 1 (PDF 422 kb)
