# Supplementary material for: Comparing Automated Morphology Quantification Software on Dendrites of Uninjured and Injured Drosophila Neurons
Source: Neuroinformatics. 2021 Aug 3;19(4):703–17. doi: 10.1007/s12021-021-09532-9 (PMC8566419; doi:10.1007/s12021-021-09532-9)
Supplement: Supplementary file 1 — (PDF 422 kb) [file 12021_2021_9532_MOESM1_ESM.pdf]

Comparing automated morphology quantification software on dendrites of uninjured and injured *Drosophila* neurons

Carolee Nguyen [1] and Katherine L Thompson-Peer [1, 2, 3,]

[1] Department of Developmental and Cell Biology, University of California, Irvine, 92697, USA

[2] Center for the Neurobiology of Learning and Memory, University of California, Irvine

[3] Reeve-Irvine Research Center, University of California, Irvine

Correspondance to: ktpeer@uci.edu

Supplementary Figures:

Figures S1, S2, and S3

Fig. S1

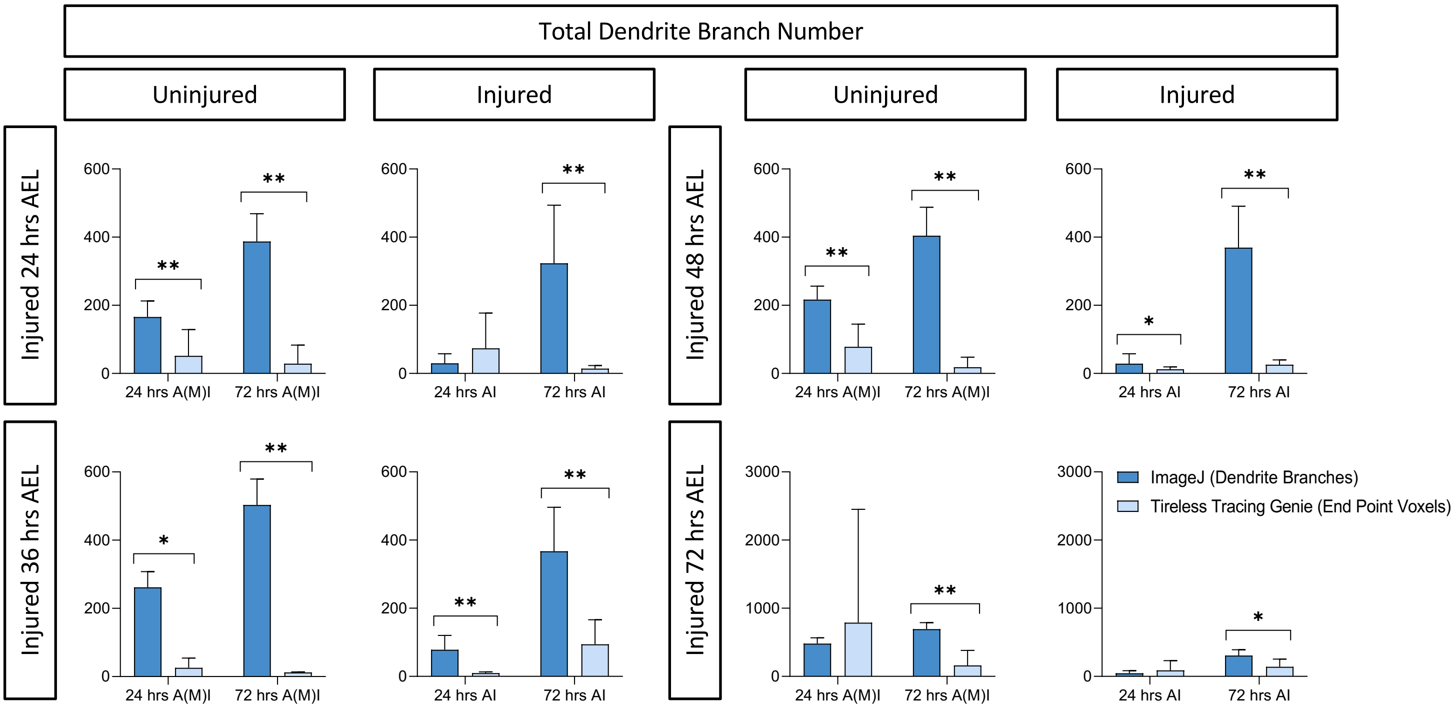

Fig S1. Tireless tracing genie consistently underestimates dendrite number.
Total dendrite tip number measured by hand tracing in ImageJ or by number of end point voxels in Tireless tracing genie is shown, ± standard deviation. * $p < 0.05$, ** $p < 0.01$ statistically significant difference by pairwise t-test. Absence of an asterisk indicates no significant difference was observed.

Fig. S2

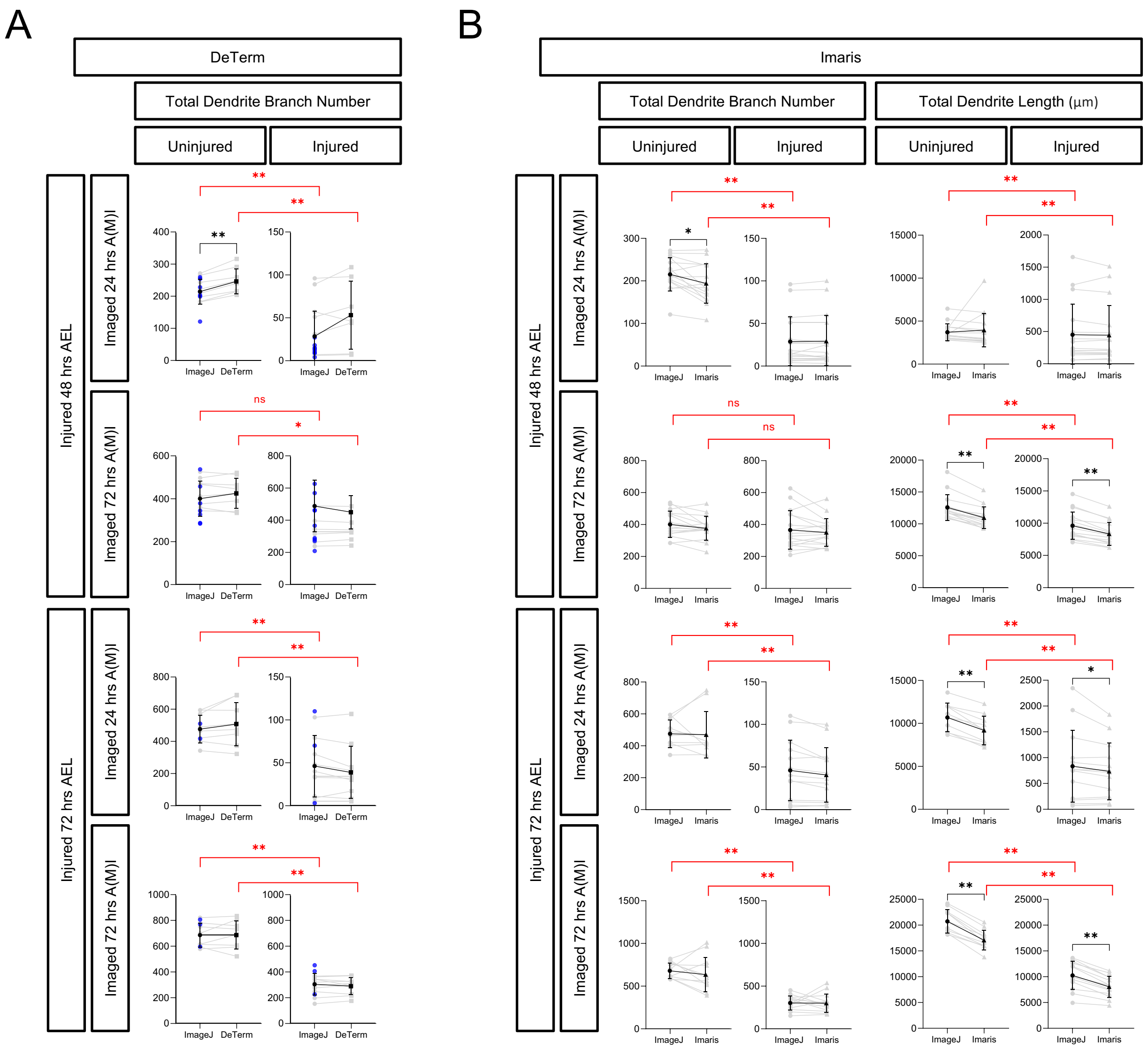

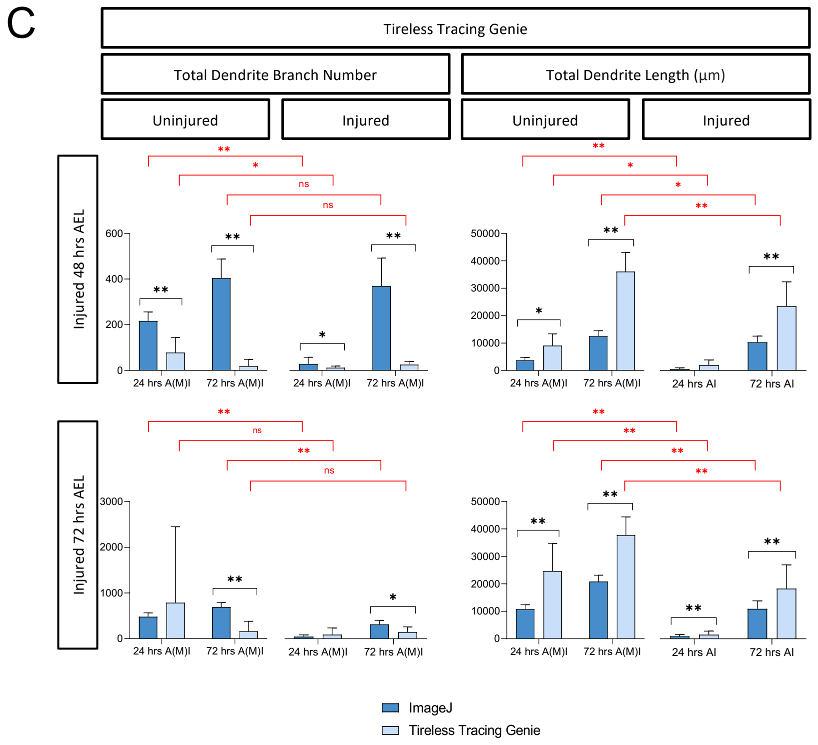

Fig S2. Individual pairwise conclusions, which are summarized in Table 2. Red brackets indicate comparisons between 24 hrs A(M)I and 72 hrs A(M)I that were significant findings of manual analysis of the data (ImageJ vs ImageJ), and the parallel comparisons of when DeTerm analyzed the same data (S2A), or Imaris (S2B), or TTG (S2C). See also Table 2 (S2A corresponds to the DeTerm column in Table 2, S2b corresponds to the Imaris column, S2C corresponds to the TTG column). A few neurons were analyzed with ImageJ but not DeTerm, these neurons are represented as blue dots in S2A. These same neurons, analyzed by both ImageJ and Imaris, are represented as gray dots in S2B.

Fig. S3

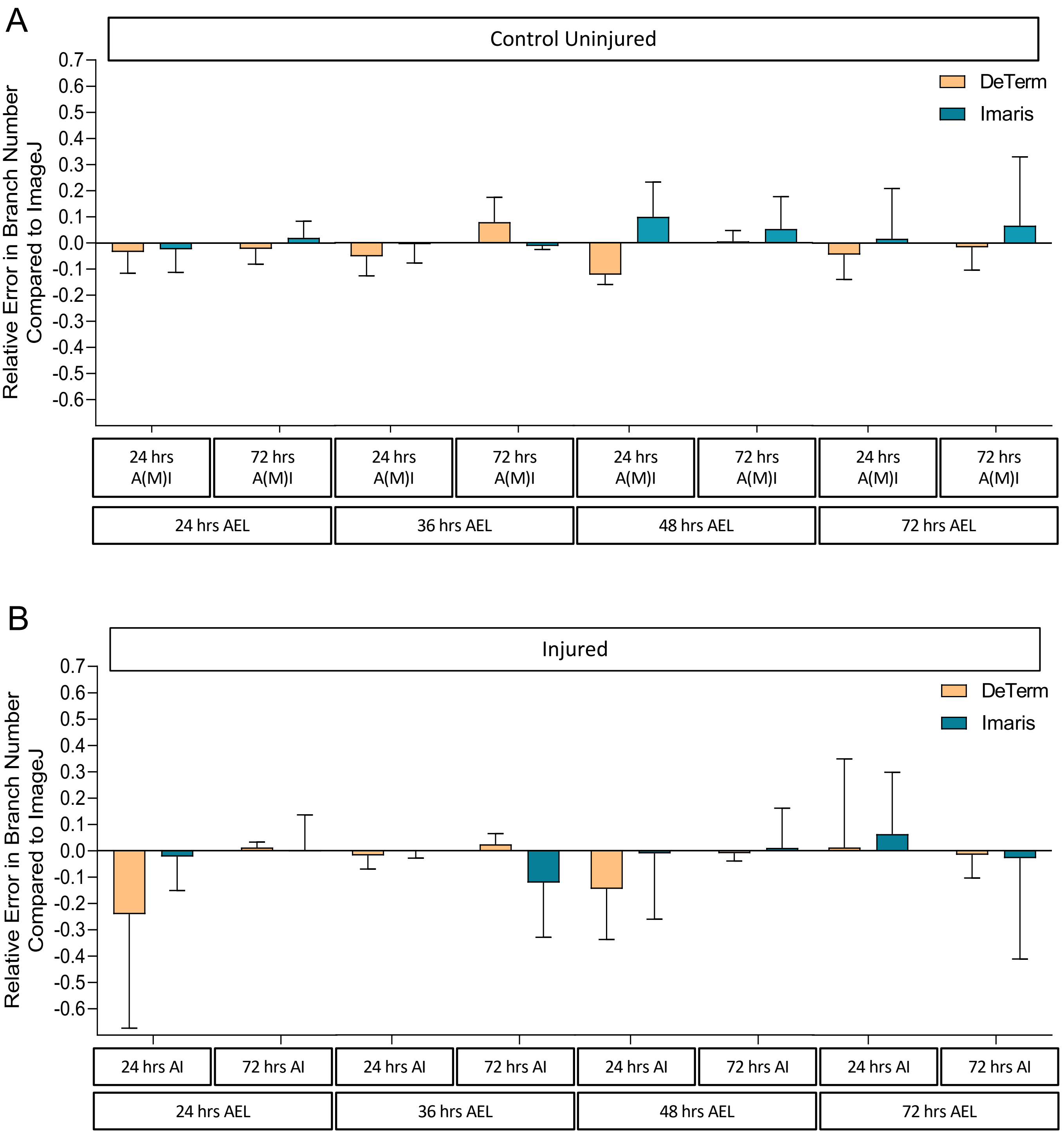

Fig S3. Relative error of DeTerm and Imaris, as compared to ImageJ and normalized to branch number. Differences in branch number were calculated by subtracting DeTerm and Imaris values of total dendrite branches and subtracting from ImageJ total dendrite branch values. These differences were normalized by averaging each value with ImageJ to reveal under and over detection of dendrite branches, for uninjured control neurons (S3A) and injured neurons (S3B). Average ± standard deviation.
